# Rapidly enlarging abdominal mass in a patient with recurrent germ cell tumor

**DOI:** 10.1002/ccr3.2476

**Published:** 2019-10-07

**Authors:** Lauren Ho, Louis Pisters, Shi‐Ming Tu

**Affiliations:** ^1^ Department of Genitourinary Medical Oncology The University of Texas MD Anderson Cancer Center Houston TX USA; ^2^ Department of Urology The University of Texas MD Anderson Cancer Center Houston TX USA

**Keywords:** oncology, urology

## Abstract

This clinical image illustrates the alarming growth rate for an embryonal carcinoma, as well as its highly curable nature. For similar cases, early diagnosis and treatment are key.

## Graphical abstract

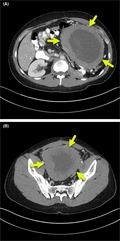

A 29‐year‐old man was diagnosed with a clinical stage IIIB nonseminomatous germ cell tumor of the testis (NSGCT). He presented with an elevated alpha‐fetoprotein level of 4533 and a retroperitoneal mass measuring 22 cm. After frontline chemotherapy comprising bleomycin, etoposide, and cisplatin (BEP) ×4, pathologic examination from a RPLND and left orchiectomy showed less than 5% teratoma with extensive necrosis in the residual lymph node.

No recurrence or metastasis was found by a CT performed 7 months later (Figure 1). After complaints of abdominal pain, nausea, vomiting, and early satiety, one month later, a CT scan showed a mass with a thick rim (Figure 2). Differential diagnoses include fibrosarcoma, Burkitt lymphoma, and parasitic abscess. Biopsy revealed embryonal carcinoma.

**Figure 1:**
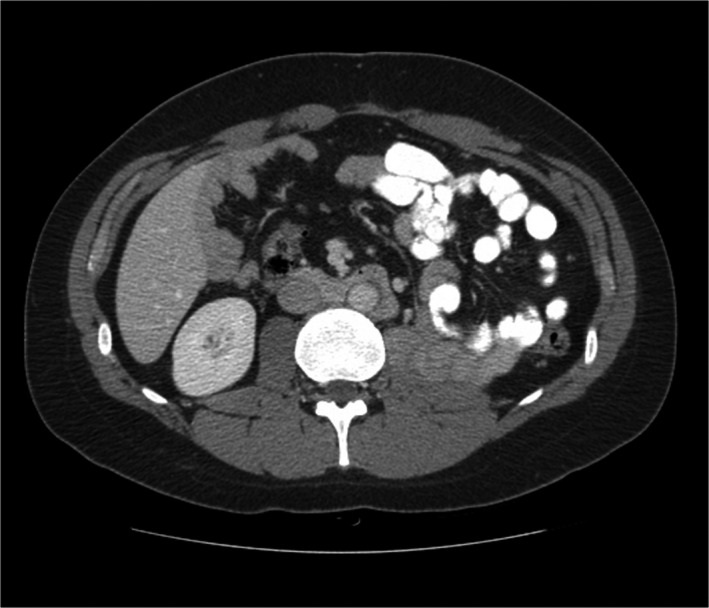
Representative image from an abdominal CT scan on 05/06/2013 showed no evidence of new mass lesion or local recurrence

**Figure 2:**
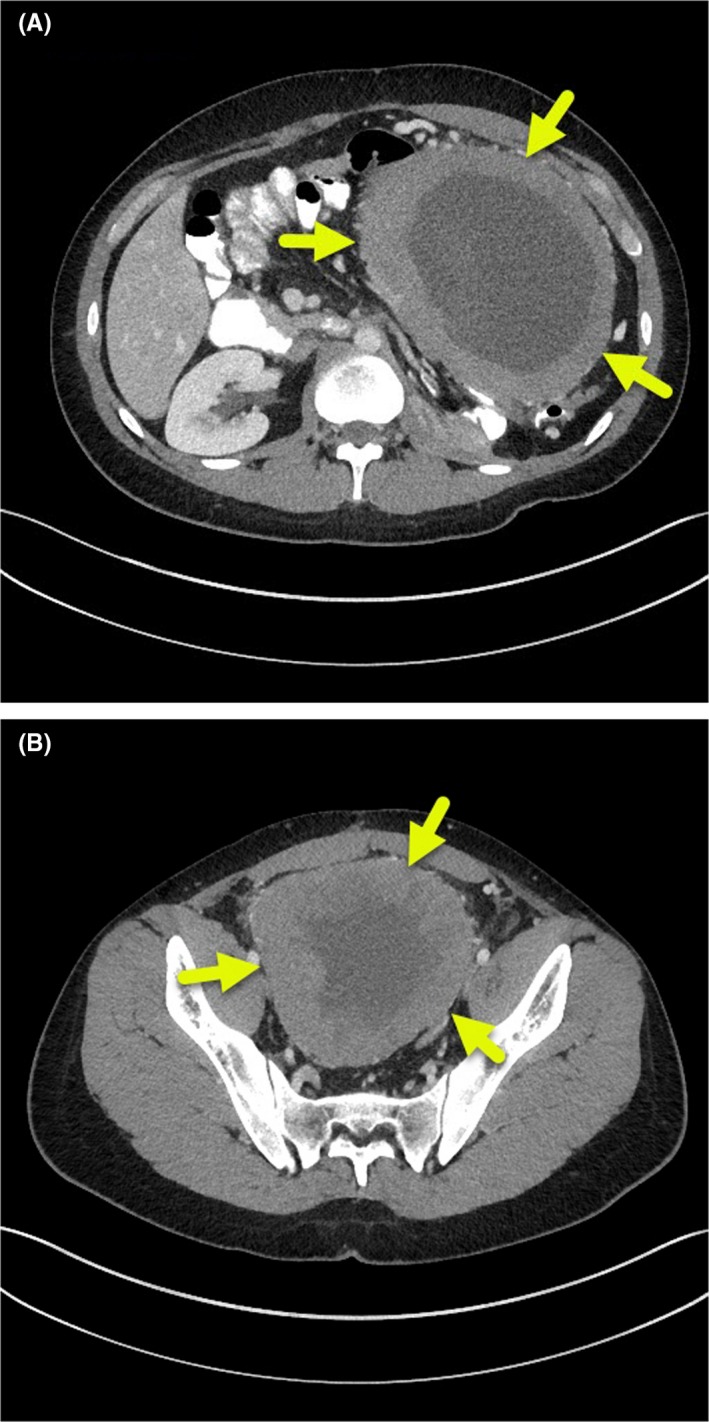
Representative image from an abdominal CT scan on 06/24/2013 showed a large, recurrent, partially necrotic abdominal pelvic mass with a thick rim (arrows)

After salvage chemotherapy comprising paclitaxel, ifosfamide, and cisplatin (TIP) ×1 and paclitaxel, doxorubicin, cisplatin (ATP) ×3, resection of the residual mass unveiled multiple minute microscopic foci of NSGCT. Four years after receiving adjuvant chemotherapy comprising TIP ×2, the patient is doing well without evidence of metastatic or recurrent disease.

To our knowledge, this is the first report of a rapidly enlarging mass in a patient with embryonal carcinoma that recurred over 7 weeks. It is highly unusual even for an isolated embryonic tumor to grow in this manner at this rate.^1^, ^2^ For men with relapsed NSGCT, a multimodality approach of chemotherapy combined with resection of all residual disease is the standard of care. Although the overall cure rate is high (>90%), early detection still offers the best potential for successful treatment.

## CONFLICT OF INTEREST

None declared.

## AUTHOR CONTRIBUTIONS

ST and LH: contributed to searching the literature, writing the report, providing relevant images, and editing the report. LP: performed the surgery. All authors have approved the final version. Written informed consent to publication was obtained.
